# Pseudoneglect during object search in naturalistic scenes

**DOI:** 10.1007/s00221-023-06679-6

**Published:** 2023-08-23

**Authors:** Antje Nuthmann, Christopher N. L. Clark

**Affiliations:** 1 Institute of Psychology, University of Kiel, Olshausenstr. 62, 24118 Kiel, Germany; 2 Psychology Department, School of Philosophy, Psychology and Language Sciences, University of Edinburgh, Edinburgh, UK

**Keywords:** Naturalistic scenes, Visual search, Eye movements, Attention, Spatial biases, Pseudoneglect

## Abstract

Pseudoneglect, that is the tendency to pay more attention to the left side of space, is typically assessed with paper-and-pencil tasks, particularly line bisection. In the present study, we used an everyday task with more complex stimuli. Subjects’ task was to look for pre-specified objects in images of real-world scenes. In half of the scenes, the search object was located on the left side of the image (*L*-target); in the other half of the scenes, the target was on the right side (*R*-target). To control for left–right differences in the composition of the scenes, half of the scenes were mirrored horizontally. Eye-movement recordings were used to track the course of pseudoneglect on a millisecond timescale. Subjects’ initial eye movements were biased to the left of the scene, but less so for *R*-targets than for *L*-targets, indicating that pseudoneglect was modulated by task demands and scene guidance. We further analyzed how horizontal gaze positions changed over time. When the data for *L*- and *R*-targets were pooled, the leftward bias lasted, on average, until the first second of the search process came to an end. Even for right-side targets, the gaze data showed an early left-bias, which was compensated by adjustments in the direction and amplitude of later saccades. Importantly, we found that pseudoneglect affected search efficiency by leading to less efficient scan paths and consequently longer search times for *R*-targets compared with *L*-targets. It may therefore be prudent to take spatial asymmetries into account when studying visual search in scenes.

## Introduction

Pseudoneglect describes the tendency to pay more attention to the left side of space (Bowers and Heilman 1980). While the phenomenon has been found in a variety of tasks (Brooks et al. 2014; Friedrich et al. 2018, for reviews), it is still mostly assessed with paper-and-pencil tasks, first and foremost by asking participants to bisect a visually presented line at its center (Jewell and McCourt 2000; McCourt 2001). Here, we investigate the degree to which pseudoneglect generalizes to a real-world task (visual search) with more complex stimuli (naturalistic scenes). We use observers’ eye movements to track how spatial asymmetries in the allocation of overt attention change over time. Moreover, we explore effects on search efficiency.

Attentional asymmetries during visual search have been investigated with different stimuli and instructions, yielding conflicting results. In classic laboratory search tasks, observers are asked to decide about the presence or absence of a target item among distractor items while holding fixation at the center of the display (Wolfe 2015). In feature-search tasks, the target is defined by the presence of a single feature, for example, a unique color (Treisman and Gelade 1980). In conjunction-search tasks, however, the target is defined by the co-occurrence of two or more features (Treisman and Sato 1990). Poynter and Roberts (2012) compared these two tasks using a within-subject design. In two experiments, performance for efficient feature search was better for left-side targets, whereas performance for inefficient conjunction search was better for right-side targets. The results were taken to support a global attentional strategy for the right hemisphere and a local attentional strategy for the left hemisphere (cf. Hellige 1996; Van Kleeck 1989). Note that the observed advantage for detecting targets on the right side in the conjunction-search task is not compatible with an attentional symmetry related to pseudoneglect.

A number of experiments used conjunction-search tasks with horizontally elongated displays, allowing for a more fine-grained manipulation of horizontal target position (Nicholls et al. 2017; Nicholls et al. 2014; see also English et al. 2021). Subjects’ task was to search for an inverted triangle among upright triangles. Nicholls et al. (2014, Experiment 4), presenting the search displays for up to 4 s, found significantly reduced error rates and faster reaction times for left-side compared with right-side targets. Nicholls et al. (2017) replicated and extended this work. In Experiment 1, the authors found an effect of target side for search times only, with the effect on error rates re-emerging for right-handers in Experiment 2. When the presentation duration of the search displays was reduced to 200 ms to counteract active exploration through eye movements, no attentional asymmetries were found (Experiment 1). The involvement of eye movements was further investigated in Experiment 3, where all displays were shown for 2 s. Reduced error rates for left-side targets were observed in the condition that required subjects to fixate centrally, but not in the free-viewing condition. Collectively, the experiments yielded inconsistent results with regard to the role eye movements play in pseudoneglect. The results further suggest that reaction times may be a more sensitive measure of attentional asymmetries than error rates (Nicholls et al. 2017).

To increase the ecological validity of the search task, other researchers have asked subjects to look for everyday objects in photographs of real-world scenes (Machner et al. 2018; Nuthmann and Matthias 2014; Pflugshaupt et al. 2004). Pflugshaupt et al. (2004) tested patients with recovered hemineglect, whereas Machner et al. (2018) tested right-hemisphere stroke patients, but both studies included a control group consisting of age-matched healthy participants.

In Pflugshaupt et al. (2004), the control group comprised 16 healthy subjects (median age: 47 years). The stimuli consisted of 32 scene images in which one of eight different search objects was placed. The examples provided in the authors’ Fig. 2 suggest that scene images were relatively symmetrical and that search objects were not contextually relevant (e.g., a clock was added to a scene depicting empty bottles). The percentage of ‘hits’ was identical for left-side and right-side targets (75%); search times were numerically shorter for left-side targets.

Machner et al. (2018) presented three patient groups and a control group with 100 different wide-screen images of writing desks that were photographed from above. In each scene, 30 everyday objects were spread across the desk. The task was to search for a paperclip (present on 80% of the trials) and to report its color (blue or red). For the 11 control subjects (mean age: 69 years), neither target detection rates (at ceiling) nor search times showed noticeable differences across four horizontal target positions.^1^

Nuthmann and Matthias (2014) used the Edinburgh scene-viewing corpus (Nuthmann and Henderson 2010; Pajak and Nuthmann 2013) to investigate the time course of pseudoneglect under different task instructions. Seventy-two neurologically healthy participants (mean age: 23 years) each viewed 135 naturalistic scenes, 45 scenes in each of three viewing tasks (memorization, preference judgment, search). Prior to each search trial, a text label described the contextually relevant search object, which was always present in the scene. A post hoc analysis yielded no significant difference in search times for target objects that were located on the left or right sides of the scene, respectively (Nuthmann and Matthias 2014).

Not finding impaired search performance for right-side objects appears to be at odds with a horizontal leftward gaze bias that has been reported in various eye-tracking studies investigating scene viewing. In typical experiments, scene exploration starts from the center of the scene, from where the first saccade is more frequently directed to the left than to the right side of the image (Dickinson and Intraub 2009; Foulsham et al. 2013; see also Müri et al. 2009; Ptak et al. 2009). Although early reports include visualizations of how the horizontal gaze bias develops over time during free visual exploration (Engmann et al. 2009; see also Pflugshaupt et al. 2004), Ossandón et al. (2014) and Nuthmann and Matthias (2014) were the first to investigate this issue systematically. They found that the leftward bias extended beyond the first eye movement and was followed by a rightward bias in free-viewing, memorization, and preference judgment tasks. During free-viewing of scenes, the initial leftward bias did not depend on image category (Ossandón et al. 2014) and was not modulated by viewing distance (Hartmann et al. 2019). For a search task, Nuthmann and Matthias (2014) found that the pseudoneglect was also present when the target object was located on the right side of the scene image.

To start searching the scene with a saccade to the left when the target object is on the right should be disadvantageous. The question then arises why previous experiments found no reduction in search performance for right-side targets (Machner et al. 2018; Nuthmann and Matthias 2014; Pflugshaupt et al. 2004). A limitation shared by these studies is that the stimulus material did not include mirror-reversed versions of the original scene images. Factors influencing search efficiency include properties related to the targets like their size and salience as well as contextual guidance (e.g., Castelhano and Heaven 2010; Malcolm and Henderson 2010; Miellet et al. 2010; Nuthmann et al. 2021). Therefore, subtle differences in attention guidance by target features or guidance by scene context can potentially mask effects of pseudoneglect on search performance. Alternatively, measures of search efficiency derived from subjects’ behavioral responses may simply be less sensitive than eye-movement records in capturing attentional asymmetries during scene search.

To distinguish between these two possibilities, we conducted an experiment in which participants had to look for a cued object that was either located on the left or right side of the scene. Scenes were used in both their original and mirror-reversed orientation (Afsari et al. 2016; Dickinson and Intraub 2009; Foulsham et al. 2013; Ossandón et al. 2014). In visual-search experiments, observers are often asked to decide about the presence/absence of the target (e.g., Machner et al. 2018; Nicholls et al. 2017; Poynter and Roberts 2012). Since we wanted to investigate how gaze becomes aligned with a designated target object, our experiment included target-present trials only (Castelhano and Heaven 2010; Malcolm and Henderson 2010; Nuthmann et al. 2021; Nuthmann and Matthias 2014; Pflugshaupt et al. 2004; Zelinsky 2008). We used a comparatively large and diverse set of scenes, in each of which one object was selected as the target. Note that this is different from previous studies in which the target was always the same (Machner et al. 2018) or in which targets were repeated (Pflugshaupt et al. 2004).

The design of our study allowed for an in-depth analysis of subjects’ initial eye movements, the central question being whether early processing of general scene information can modulate the initial pseudoneglect during scene search. A second goal was to examine the time course of pseudoneglect during scene search (Nuthmann and Matthias 2014) with a refined analysis method for which the gaze raw data were used. To investigate the manner in which the oculomotor system compensates for the pseudoneglect bias, we provide additional time-course analyses considering basic characteristics of saccades and fixations. Critically, we revisit the question whether pseudoneglect affects behavioral and eye-movement measures of search efficiency.

## Methods

### Participants and apparatus

Twenty-two participants (mean age: 32.5 years; 11 women, 11 men) with normal or corrected-to-normal vision took part in the study. Eighteen participants reported to be right-handed (4 left-handed, all women). All participants gave their informed consent before the experiment. The Psychology Department at the University of Edinburgh granted ethics approval for the study, which conformed to the Declaration of Helsinki.

Stimuli were presented on a 21-inch CRT monitor at a viewing distance of 90 cm, with each scene image subtending a visual angle of 25.78° horizontally × 19.34° vertically. The experiment was implemented in SR Research Experiment Builder. Participants used a four-button Microsoft Sidewinder controller to indicate that they had found the target object.

Eye movements were recorded with an SR Research EyeLink 1000 Desktop mount system, which was equipped with the 2000 Hz camera upgrade, allowing for binocular recordings at a sampling rate of 1000 Hz for each eye. A chin rest with head support minimized head movement. We placed the chin rest such that the participant’s midpoint between the centers of the eyes was aligned with the vertical midline of the screen. We adjusted table height to place the participant’s straight-ahead view at the midpoint of the screen.

### Procedure

To investigate visual search in naturalistic scenes, a target acquisition task was used (Zelinsky 2008). Participants were instructed to search each scene for a pre-specified target object. Once they had found the target, participants should look at it and press any button on the controller (e.g., Castelhano and Heaven 2010; Nuthmann and Malcolm 2016).

The experiment started with a 9-point calibration of the eye tracker, followed by a validation; this procedure was repeated as required thereafter. Participants were shown four practice trials to familiarize themselves with the task. The main experiment consisted of 148 trials spread across three blocks (50 scenes, 50 scenes, and 48 scenes), with a break between each block. For both the practice and the experimental trials, scene order was randomized.

The trial structure was as follows: central fixation cross; word identifying the search target; central fixation cross; scene image. A custom gaze-contingent implementation checked whether the eyes fixated in an area of 1.93° around the cross for a duration of 600 ms. If participants failed the first check, a new calibration/validation routine was initiated. The target object name was presented at the center of the screen for 1.5 s. The search scene was displayed until the participant pressed a button on the controller or until 15 s had passed. The inter-trial interval was 250 ms.

### Design and stimuli

To investigate left–right asymmetries, the factor target location (left vs. right) was manipulated within participants and within scene items. The main stimuli consisted of 148 colored photographs of complex real-world scenes (800 × 600 pixels). Eighty-seven of these images were taken from the Edinburgh scene-viewing corpus (e.g., Nuthmann and Henderson 2010; Nuthmann and Matthias 2014) and other object-in-scene search experiments (Nuthmann 2014; Nuthmann and Malcolm 2016). An additional 61 scenes were obtained by student research assistants. The selection of scenes ensured that the images did not include cues that could make subjects aware of the fact that some of the scene images were mirror-reversed (Ossandón et al. 2014). Most of the images (*n* = 122) depicted indoor scenes from different categories.

Each scene image contained a contextually relevant search target with an average size (width × height) of 3.17° × 3.29°. In half of the scenes, the search object was located on the left side of the image (*L*-target); in the other half of the scenes, the target was on the right side (*R*-target). The shortest distance between object center and the center of the scene image was not significantly different for left-side and right-side targets, *t*(146) = −0.55, *p* = 0.581. The same was true for the shortest distance between object center and the vertical midline, *t*(146) = −0.21, *p* = 0.836.

**Fig. 1:**
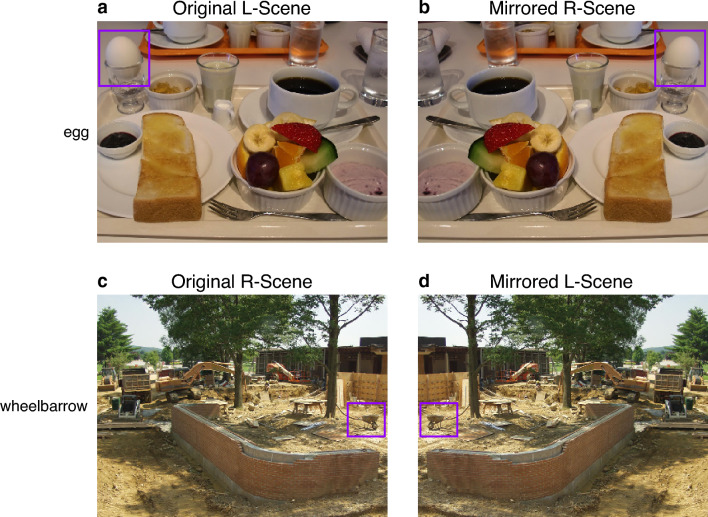
Example scenes used in the study and their search targets. In the original scenes, the target was either located on the left side of the image, as shown in panel **a**, or on the right side, as depicted in panel **c**. Scenes were mirror reversed to create an *R*-scene corresponding to each original *L*-scene, shown in **b**, and an *L*-scene for each original *R*-scene, shown in **d**. In each panel, the violet rectangle denotes the AOI encompassing the target object

Each of the 74 *L*-scenes and 74 *R*-scenes was flipped along the vertical axis, creating an *R*-scene for each original *L*-scene, and an *L*-scene for each original *R*-scene (Fig. 1). Figure 7 shows the distribution of all original and mirror-reversed search objects. Target location (left vs. right) and stimulus orientation (normal vs. mirror-reversed) were fully crossed and counterbalanced across participants. Participants viewed each scene only once over the duration of the experiment, and the search target was equally likely to appear on the left or right side of the scene image.

### Data analysis

The gaze raw data were processed with SR Research Data Viewer. We created a sample report for the time-course analysis, and a fixation report for all other analyses of the eye-tracking data. The eye tracker’s cognitive configuration (i.e., the default) was used to detect saccades, blinks, and fixations. The reports were further processed with R and MATLAB (The MathWorks, Natick, MA). Figures were created with the *ggplot2* R package (version 3.4.1, Wickham 2016) or MATLAB.

No data were recorded for one of the trials, leaving 3255 trials for analysis. In 2% of trials (*n* = 64), no response was made within 15 s. A response was scored as correct if the position of the left eye, the position of the right eye, or the average position of both eyes was within the area of interest (AOI) comprising the target during the button-press response. The AOI was a rectangular box that was closely drawn around each target object, with 0.75° of padding added to the target area. Correct responses were obtained for 91.9% of the trials (*n* = 2990). Only correct trials were included in the reaction-time and eye-movement analyses. Data from the right eye were used for analyses involving oculomotor measures, unless otherwise stated.

For analyses of the eye-movement data, a number of data exclusion criteria were applied. Fixations with off-screen coordinates were removed. Fixations and saccades that co-occurred with blinks were also excluded. Similarly, invalid data samples were excluded from analyses involving the raw gaze data. For analyses of fixation durations, we further excluded fixations that were the last fixation in a trial and fixations during which the button press took place (typically, but not always, this fixation was also the last fixation in a trial). The first fixation in a trial was not considered when comparing mean fixation durations for the two experimental conditions; this initial fixation was, however, included in a time-course analysis of fixation durations.

To quantify the pseudoneglect and to evaluate its effects on search performance, the behavioral and eye-movement data were analyzed with linear mixed-effects models (LMM, Baayen et al. 2008) or generalized linear mixed-effects models (GLMM, Jaeger 2008). For binary data, binomial GLMMs with a logit link function were used. Continuous response variables (e.g., search times) were analyzed with LMMs. Data were analyzed at the level of individual observations, that is without prior averaging. Search times and fixation durations were analyzed both log-transformed and untransformed, with no qualitative differences in results, which is why we report the fixed-effect estimates on the original scale of measurement.

To assess left–right asymmetries, the mixed models included the two-level factor ‘target location’ as fixed effect. For contrast coding, the default was to use dummy coding with ‘left side’ as the reference level. Therefore, the intercept for the fixed effect ‘target location’ estimates the mean response for scenes in which the target was located on the left side. The slope estimates the difference between right and left target locations. The predicted response for right-side targets can therefore be obtained by adding the difference score to the intercept. Deviations from the default coding scheme are described in individual sections below.

All mixed models included by-subject random intercepts and by-item random intercepts. Random slopes were included if they were justified by the design (Barr et al. 2013) and retained if they were supported by the data (Matuschek et al. 2017).

(G)LMM were fit to the data using the *(g)lmer* program of the R *lme4* package (version 1.1–27.1, Bates et al. 2015) with the default settings. For LMMs, *p* values for fixed effects were obtained using Satterthwaite approximation as implemented in the *lmerTest* R package (version 3.1–3, Kuznetsova et al. 2017).

## Results and discussion

The results are presented in four subsections. First, we investigate participants’ initial orienting by analyzing properties of the first saccade that occurred after scene onset. Next, we use the raw gaze data to examine how spatial asymmetries develop over time during scene search. This pseudoneglect analysis is complemented by additional time-course analyses of saccade directions and amplitudes as well as fixation durations. Finally, we investigate the degree to which pseudoneglect affects search efficiency.

### First saccade

Within the scene stimuli, the target object was either located on the left side or on the right side. For the subject, ‘left’ and ‘right’ may be defined with respect to the trunk, the head, or the eyes (see Colby 1998). The experimental setup was chosen such that *L*-targets appeared in the left hemifield and *R*-targets in the right hemifield, represented in body-centered coordinates. In eye-centered coordinates, however, the left–right position of the search object is defined with respect to the current eye fixation. The initial central fixation at the beginning of each experimental trial therefore constitutes a special case where the fixed trunk- and head-centered reference frames more or less align with the otherwise dynamic eye-centered spatial reference frame.

Starting from the center of the scene image, observers could make a first saccade in any direction. Results from existing scene-viewing studies, however, suggest that the first saccade is more frequently directed to the left than to the right side of the image (Dickinson and Intraub 2009; Foulsham et al. 2013). Moreover, there is a general preference for making saccades to the left or right compared with saccades in any other direction during scene exploration (horizontal bias, Foulsham and Kingstone 2010; Foulsham et al. 2008; Van Renswoude et al. 2016). For the first saccade within the scene, Foulsham et al. (2018) found that the horizontal bias was modulated by the pseudoneglect bias (or left-bias), such that horizontal saccades were more frequently directed to the left than to the right.

Here, we explore the degree to which these findings extend from scene viewing to visual search in scenes. Object-in-scene search is a task during which top–down influences on attention and eye guidance are known to dominate (Koehler et al. 2014; Malcolm and Henderson 2010). During the initial central fixation, observers can retrieve information about the overall meaning and spatial layout of the scene, providing some indication about where the cued search object is likely to be found (Eckstein et al. 2006). Can such early scene processing modulate the early left-bias during scene search? If so, we should observe fewer left-directed first saccades when searching for right-side compared with left-side targets.

**Fig. 2:**
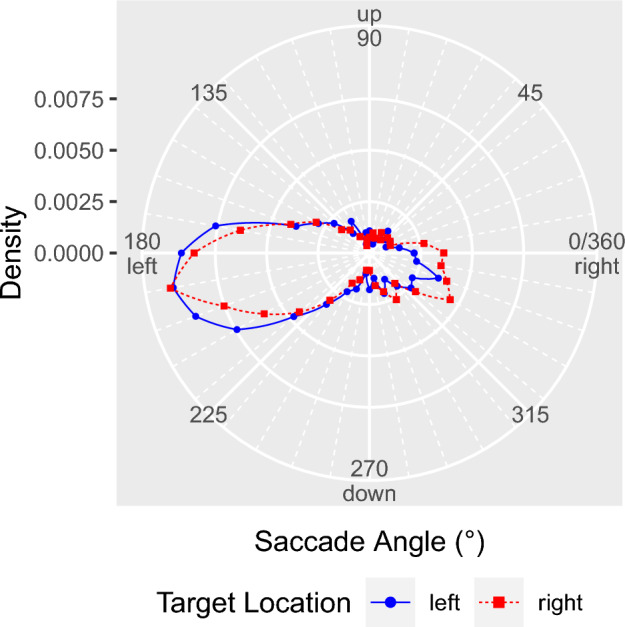
Direction of the first saccade. Distribution of saccade directions when searching for left-side targets (blue dots connected by a solid line) and right-side targets (red squares, dashed line) within scenes. The polar histograms depict densities and were constructed using a bin size of 10°. The dots and squares are located on radial grid lines that represent the 36 bin centers

To test this hypothesis, we analyzed initial eye movements with an amplitude larger than 1° (Foulsham et al. 2013, 2018), which were launched from within the scene’s central region. The direction of the first saccade was measured as the angle between the horizontal plane and the line connecting the initial central fixation with the endpoint of the first saccade. For each of the two target-location conditions, a polar histogram was constructed by sorting saccade angles from all observers into 36 equally spaced bins of 10° (Fig. 2). For both *L*-targets and *R*-targets, there was a bias to direct the first saccade to the left side of the scene. This left-bias was somewhat less pronounced when the search target was located on the right side of the scene.

The density histograms in reveal an additional tendency to direct the first saccade to the lower quadrants of the scene. Across scenes, more targets appeared in the lower half compared with the upper half of the scene (Fig. 7). Therefore, the slight downward bias in saccade directions (Fig. 2) may indicate a tendency for first saccades to take the eyes in the direction of the search target in the scene. Nevertheless, when the target was on the right, the first saccade was biased toward the left. Collectively, the data suggest that the leftward and downward biases independently influenced the programming of the saccade’s direction.

**Fig. 3:**
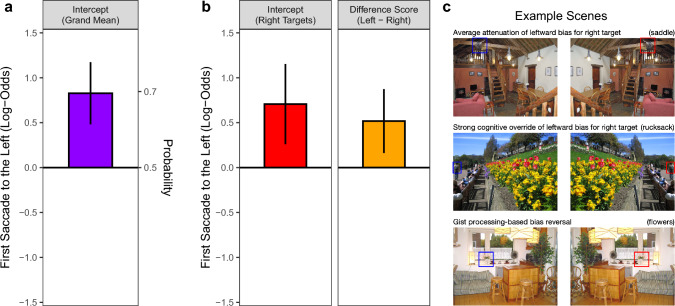
Probability of the first saccade going to the left. **a** The overall probability was evaluated with a binomial varying-intercept GLMM with no predictors. The height of the violet bar represents the fixed-effect estimate for the intercept on the log-odds scale. The right *y*-axis indicates probability values; the error bar represents the 95% confidence interval (CI =  ± 1.96 × SE). **b** The effect of target location on the left-direction of the first saccade was assessed with a dummy-coded GLMM including a by-item random slope for ‘target location’. The red bar represents the fixed-effect estimate for the right targets, whereas the orange bar depicts the difference between left and right targets. **c** The bar charts in **b** are complemented by three example scene pairs for which the results were either similar or dissimilar to the mean response. See text for details

For statistical analysis, the direction of each saccade was dichotomized into left (> 90° and < 270°) or right (< 90° or > 270°). If there was a reliable directional preference for saccade targets in the left hemispace, the probability of the first saccade going to the left should be significantly larger than 0.5. We tested this hypothesis with a binomial varying-intercept GLMM with no predictors. The model included the intercept as a fixed effect and allowed it to vary by subject and scene. The parameter estimates were obtained on the log-odds or logit scale, with a logit of 0 corresponding to a probability of 0.5. Consequently, if the fixed-effect estimate for the intercept is significantly different from zero, the null hypothesis of no directional preference for the first saccade can be rejected. The intercept was significantly larger than zero, *b* = 0.83, *SE* = 0.18, *z* = 4.69, *p* < 0.001, with a logit value of 0.92 corresponding to a probability of 0.70 (Fig. 3a). Thus, the very first saccade went more often to the left than to the right side of the image.

In addition, we tested whether the probability of the first saccade going to the left was modulated by target location. To this end, the dummy-coded factor ‘target location’ was added as fixed effect to the GLMM, with *R*-targets serving as the reference level. The model included a by-item random slope for ‘target location’. A by-subject slope was not included, due to a lack of variance in the data. The fixed-effect estimate for *R*-targets was significantly larger than zero, *b* = 0.71, *SE* = 0.23, *z* = 3.09, *p* = 0.002 (Fig. 3b). The probability for making a left-directed first saccade was significantly increased for *L*-targets compared with *R*-targets, *b* = 0.52, *SE* = 0.18, *z* = 2.81, *p* = 0.005.

The random-slope model provided a significantly better fit to the data than the model including random intercepts only, logLik Δχ^2^(2) = 165.4, *p* < 0.001. The considerable improvement in goodness of fit indicates that the scene items varied considerably in their response to the left–right manipulation of the target’s location. To explore this variability, we visualized the conditional modes of the by-item random effects from the dummy-coded GLMM and from recoded models using simple coding (-0.5/ + 0.5). This way, we identified two types of deviations from the fixed-effect estimates, which will be discussed with reference to example scenes.

The target in the outdoor scene depicted in Fig. 3c was a rucksack on the back of a man walking through Princes Street Gardens. The composition of the scene strongly constrained the search space to one side of the image, with observers often directing their first saccade to that side. For targets on the right, this translates to a strong cognitive override of the pseudoneglect bias. Scene-gist processing during the initial central fixation sometimes even led to a bias reversal, due to competition between semantically related objects. For example, this happened when the task was to search for flowers in a living room scene (Fig. 3c, bottom row). The flowers were in a vase that was close to the center of the scene. However, the target was neither particularly large nor visually salient. Moreover, the scene contained a large green plant on the side opposite to the flowers. When searching this scene, the first saccade often went to the side of the plant, leading to a rightward bias when the flowers were on the left and a leftward bias when the flowers were on the right.

### Time course of pseudoneglect

When searching for objects in naturalistic scenes, observers typically make more than one saccade to acquire the target. Thus, the eye-movement behavior accompanying the search process unfolds over time. For each search trial, the eye tracker provides a raw gaze trace, which is subsequently parsed into a sequence of fixations and saccades (Fig. 8, left panels). Our main time-course analysis was based on the raw gaze data as they provided us with the most precise measurement of how the allocation of attention and gaze changes over time. For each millisecond in a given trial, we retrieved the horizontal deviation of gaze from the vertical midline of the image (Fig. 8c). Gaze positions on the left/right side of the scene, therefore, have negative/positive horizontal deviations. Given that the two eyes are not always perfectly aligned (Kirkby et al. 2008), we analyzed average binocular gaze positions (i.e., we took the average across the two eyes).

**Fig. 4:**
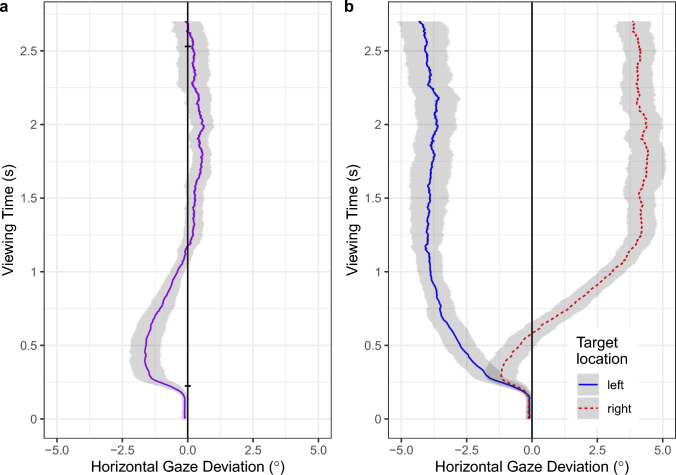
Time course of pseudoneglect during object-in-scene search. Spatial asymmetries are described as a change of horizontal gaze deviation (°) over time. The vertical line at 0° represents the vertical midline of the scene, with negative deviations indicating a leftward bias in the distribution of attention and gaze. **a** Combined data for all scene images. The two small horizontal lines intersecting the vertical midline mark the mean duration of the initial central fixation and the mean search time, respectively. **b** The data are depicted separately for scenes in which the target was located on the left (blue solid line) or right (red broken line) side of the image. In both panels, the shaded area around the mean depicts a 95% bootstrap confidence interval. See text for details

In the first analysis, the data from the *L*- and *R*-scenes were pooled. For each subject, their trial-based curves were averaged. The subject-based curves were then averaged across subjects (Fig. 4a).^2^ A mean deviation of zero means that the deployment of attention and fixation was symmetric at this point in time. Negative deviations are indicative of a leftward bias in the distribution of attention. The data in Fig. 4a show how the horizontal deviation of gaze changed during the search process. During the initial central fixation, the mean horizontal gaze deviation was effectively zero. As the eyes prepared their first saccade away from the center of the scene, an initial leftward bias developed, which lasted until the first second of scene exploration came to an end. The peak deviation was close to −2°. The early leftward bias was followed by a weak rightward bias later in time. Toward the end of the search process, the mean horizontal gaze deviation returned to zero. This is to be expected, because the mean distance between object and image center was the same for left-side and right-side targets (see Methods).

In the next analysis, we contrasted scenes in which the target was located on the left vs. right side of the image (Fig. 4b). Unsurprisingly, gaze positions on scenes with left-side targets showed a sustained bias toward the left side of space (blue solid line). Importantly, this initial leftward bias was also present when the search object was located on the right side of the image (red broken line).

### Additional time-course analyses

Parsing the raw gaze data from each search trial into a sequence of oculomotor events gives rise to a scan path (Holmqvist et al. 2011). Spatial and temporal aspects of scan path properties are well described by the direction and amplitude of a given saccade and the duration of the subsequent fixation. An extensive body of research has shown that fixation durations increase during initial viewing periods and stabilize during later viewing (see Pannasch et al. 2008, for a review). Conversely, saccade amplitudes first increase and then reach a plateau, before decreasing again during later viewing periods. The pattern has been observed for various visual-cognitive tasks ranging from visual search in simple displays (Over et al. 2007; Scinto et al. 1986) to the exploration of naturalistic scenes under different viewing instructions, including visual search (Castelhano et al. 2009; Mills et al. 2011; Unema et al. 2005).

**Fig. 5:**
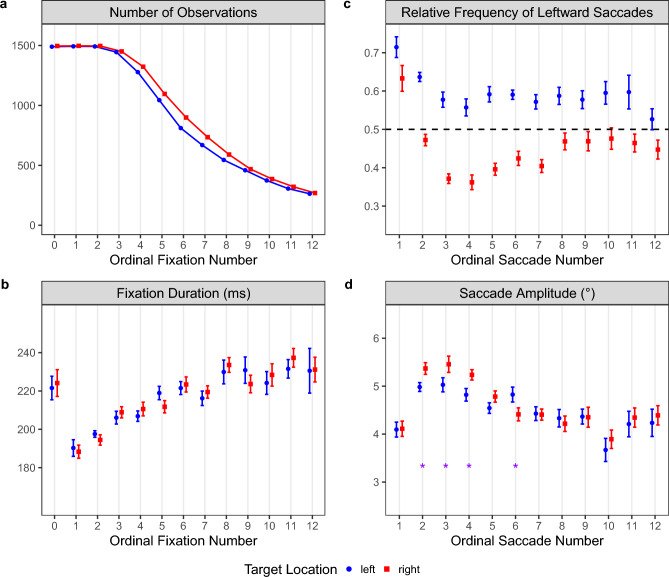
Adjustment of eye-movement parameters over time depending on whether the target object was located on the left side (blue dots) or right side (red squares) of the scene. **a** Total number of observations for the initial central fixation (#0) and the following 12 fixations. **b** Mean fixation duration for each ordinal fixation. **c** Mean relative frequency of leftward saccades, with values above the dashed line indicating that more saccades went left than right. **d** Mean saccade amplitude for each of the first 12 saccades, with the asterisks indicating a significant difference between the two target-location conditions. In panels **b**, **c,** and **d**, the means were computed across participants’ means, with the error bars representing within-subjects standard errors (Cousineau 2005; Morey 2008). Note that the *y*-axes in these panels do not start at zero

Here, we analyzed how mean fixation durations, saccade amplitudes, and saccade directions change over time to explore how the oculomotor system responds to the pseudoneglect bias during object search in scenes. Accordingly, the data were analyzed separately for the two target-location conditions (Fig. 5). To depict the time course, fixations and saccades were analyzed with respect to their ordinal position in the scan path. Given that fast searches are associated with fewer saccades and fixations than slow searches, the number of available observations decreases over time (Fig. 5a). Fixation #0 represents the initial central fixation, which started prior to the onset of the scene image and ended with the execution of the first saccade. In the present context, the duration of fixation #0 was calculated as the time between scene onset and the onset of the first saccade. Note that fixation #0 is typically excluded from time-course analyses. Leaving this fixation aside, fixation duration systematically increased over time until reaching asymptote (Fig. 5b), which is consistent with previous research (e.g., Castelhano et al. 2009; Mills et al. 2011; Nuthmann 2017). For a given ordinal fixation number, mean fixation duration did not differ between searches for left-side and right-side targets.

As can be seen in Fig. 5d, saccade amplitudes show the typical pattern of increase, plateau, and then decrease (e.g., Mills et al. 2011; Over et al. 2007). The first saccade had a mean amplitude of about 4° in both target-location conditions. The second, third, and fourth saccades, however, were longer in amplitude for right-side than for left-side targets (Fig. 5d). On trials with right-side targets, the probability of making a left-directed first saccade was greater than chance (Fig. 3b). Subsequent saccades were, however, more often directed to the right than to the left (Fig. 5c). Together, the results for right-side targets indicate that the oculomotor system responded to suboptimal first-fixation locations on the left side of the scene by reversing the direction and lengthening the amplitude of some of the following saccades.

**Fig. 6:**
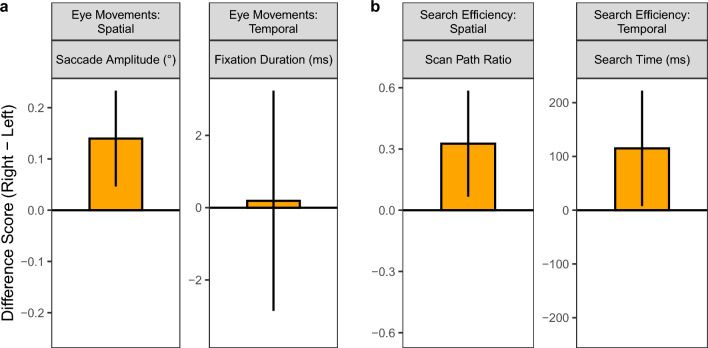
The effect of target location on **a** spatial and temporal eye-movement measures and **b** measures of search efficiency. In the linear mixed models, the factor ‘target location’ was dummy coded, with the left targets being the reference level. In a given panel, the zero line therefore represents the left targets, with the orange bar depicting the difference between right and left targets. Error bars are 95% confidence intervals, which means that the effect of target location is significant when the error bar does not include 0

**Fig. 7:**
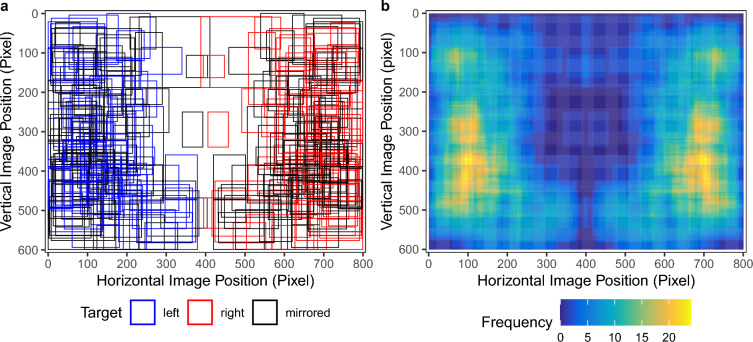
**a** Distribution and size of bounding boxes for 74 left-side targets (blue), 74 right-side targets (red), and 148 mirror-reversed targets (black). **b** Pixel-based object map representing the distribution of all search objects across all scenes

Across all ordinal positions in the scan path, the estimated saccade amplitude was *b* = 4.49 (*SE* = 0.08, *t* = 55.6, *p* < 0.001). Saccade amplitudes were significantly longer in trials with *R*-targets than in trials with *L*-targets, *b* = 0.14, *SE* = 0.05, *t* = 2.93, *p* = 0.003 (see Fig. 6a). Mean fixation duration, however, did not differ between searches for right-side and left-side targets, *b* = 0.19, *SE* = 1.55, *t* = 0.12, *p* = 0.903.

### Effects of pseudoneglect on search performance

Do the observed spatial asymmetries affect search performance? Search accuracy was high (see above), with results from a binomial GLMM indicating that there was no significant difference between left and right targets (*b* = 0.05, *SE* = 0.13, *z* = 0.38, *p* = 0.706).

**Fig. 8:**
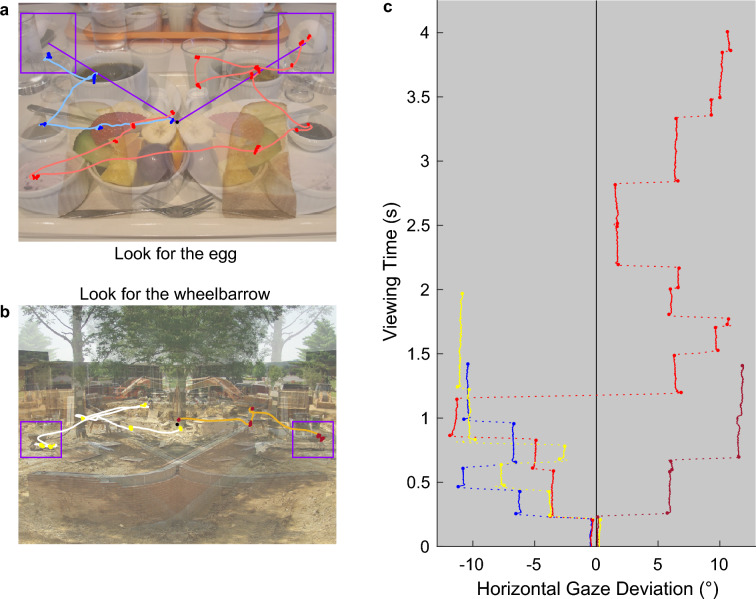
Examples visualizing how individual two-dimensional gaze traces were converted to changes of horizontal gaze deviation over time. **a** Raw gaze data obtained for the *L*-scene and the corresponding mirrored *R*-scene displayed in the top row in Fig. 1. The data are plotted over a blended version of the two images. The blue gaze trace is from an observer searching for the target object (the egg) in the upper left of the image. Correspondingly, the red gaze trace leads to the mirrored target object in the upper right. Saccades are plotted in lighter colors than fixations. The violet rectangles denote the interest areas comprising the target objects. In addition, the straight violet lines connect the center of the image (black dot) with the center of the respective interest area, thereby representing the most direct path to the target object. **b** Gaze traces obtained for the *R*-scene and the mirrored *L*-scene that are shown in the bottom row of Fig. 1. The gaze trace to the right-side target is presented in brown (fixations) and orange (saccades), and the one to the left-side target in yellow (fixations) and white (saccades). **c** The four gaze traces from the left panels displayed as horizontal gaze deviation from the vertical midline of the scene. The line colors are the colors in which the fixations appear in the left panels. The solid vertical segments are the fixations, and the dotted horizontal lines are the saccades. Additional dot markers highlight the beginnings and ends of saccades. Both fixations and saccades were included in the averaging process described in the text

However, the observed left-bias may adversely affect observers’ efficiency in correctly locating targets on the right side of the scene. To test this, we analyzed both a spatial and a temporal measure of efficiency (Fig. 6b). Scan path ratio (SPR) is a spatial measure reflecting the efficiency of eye-movement guidance to the target (Henderson et al. 1999). SPR is defined as the ratio of the path the eyes took to fixate the target divided by the most efficient possible path (see Eq. (1) in Brockmole and Henderson 2006). A measure for the path taken is obtained by adding up the amplitudes of all saccades made before first fixating the target object. The most efficient path can be approximated as a straight line from the initial fixation position at image center to the center of the target object (see Fig. 8a). An SPR of 1 indicates a direct path to the target, with values greater than 1 indicating increasingly inefficient paths. For search targets that were positioned on the left side of the scene image, the estimated SPR was *b* = 3.25 (*SE* = 0.18, *t* = 17.78, *p* < 0.001). Interestingly, SPR was significantly larger for right than for left targets (*b* = 0.33, *SE* = 0.13, *t* = 2.46, *p* = 0.023, Fig. 6b).

An important question to address is whether the less efficient scan paths for *R*-targets led to longer search times for these targets. For *L*-targets, the estimated search time was *b* = 2518 ms (*SE* = 143, *t* = 17.65, *p* < 0.001). Search times were significantly longer for *R*-targets compared with *L*-targets (*b* = 115, *SE* = 55, *t* = 2.10, *p* = 0.036, Fig. 6b).

## General discussion

Patients with left hemispatial neglect and neurologically healthy individuals both show asymmetries in the allocation of attention. Patients experiencing left neglect show a marked bias consisting of enhanced attention to the right (Bartolomeo and Chokron 2002; Cox and Aimola Davies 2020). In contrast, neurotypical individuals bias their attention slightly toward the left hemispace, thereby ‘neglecting’ rightward features of the stimulus (Jewell and McCourt 2000). We investigated this pseudoneglect (Bowers and Heilman 1980) during visual search, a task that has been used extensively to study the deployment of attention (Lauer and Võ, 2022; Wolfe 2015).

In the experiment, participants looked for objects in naturalistic scenes while we recorded the extent to which they actively explored the scenes via eye movements. Previous research on eye guidance during scene perception and search has identified a number of viewing biases: the central fixation bias (Tatler 2007), the horizontal saccade bias (Foulsham and Kingstone 2010; Foulsham et al. 2008), and a bias for the eyes to keep moving in the same direction (Smith and Henderson 2009). The pseudoneglect bias presents a relatively new addition to this list (Nuthmann and Matthias 2014; Ossandón et al. 2014).

Here, we used a target acquisition task to direct participants’ attention toward a predefined target object, which was either on the left or right side of the scene. Our data confirm that pseudoneglect modulates the horizontal bias for the first saccade, which went more often to the left than to the right (Figs. 2, 3a, see also Foulsham et al. 2018). A new finding was that, on average, this left-bias was less pronounced when the target object was on the right (Fig. 3b), indicating that pseudoneglect was modulated by contextual information generated from the initial central fixation.

Moreover, the mixed-model results suggested that our scene items varied in the extent to which the left–right manipulation of the search target’s location affected the direction of the first saccade within the scene. In particular, some of the scene stimuli showed a cognitive override of the leftward bias for right-side targets (Fig. 3c). This particular finding bears some similarity to results from studies in which a painful electrical stimulation (Schmidt et al. 2018) or a nonpainful tactile stimulation (Ossandón et al. 2015) was applied to a subject’s left or right hand during free-viewing of naturalistic scenes. In these studies, the direction of the first saccade was biased toward the site of the stimulation.

In addition to the first saccade, we analyzed how horizontal gaze positions changed over time. When the data for *L*- and *R*-targets were pooled, the averaged raw gaze data showed a distinct early leftward bias (Fig. 4a). This bias was present even when the search object was located in the right hemispace (Fig. 4b), replicating Nuthmann and Matthias (2014). Concretely, the average response to *R*-targets consisted of an initial ‘detour’ to the left, which was followed by a gradual rightward shift in horizontal gaze position.

To illuminate the underlying adjustments in oculomotor behavior, we explored how basic eye-movement parameters changed over time, separately for the two target-location conditions (Fig. 5). When the target is on the right, a straightforward way to compensate for the initial leftward bias is to make a relatively long saccade to the right. We found that the saccades following the first one did show such adjustments in amplitude and direction. Apart from that, the data presented in Fig. 5 replicate previous findings showing that observers adjust their fixation durations and saccade amplitudes over the course of scene viewing and visual search (e.g., Castelhano et al. 2009; Mills et al. 2011). Specifically, short fixations and long-amplitude saccades during early viewing were followed by longer fixation durations and shorter saccades during later viewing.

With the present study, we also wanted to clarify whether the observed attentional asymmetries affect search performance. Finding common objects in naturalistic scenes is a fairly easy task (Biederman et al. 1973). On the majority of trials, participants successfully located the target in the scene, with no disadvantage for targets on the right side. For correct trials, mixed-model analyses showed that the initial leftward bias was associated with less efficient scan paths to the target if the target was located on the right side of the scene compared with the left side. Consequently, button-press response times were significantly longer for right-side than for left-side target objects. We note that, although the reported effects were statistically significant, they were small in size (Fig. 6b).

On the one hand, our results support the view that reaction times may be a more sensitive measure of attentional asymmetries than error rates (Nicholls et al. 2017). On the other hand, finding significantly longer search times for *R*-targets than for *L*-targets differs from previous studies in which no such reduction in search efficiency was observed (Machner et al. 2018; Nuthmann and Matthias 2014; Pflugshaupt et al. 2004).

Whereas our scene stimuli were presented in their original and mirror-reversed orientation (e.g., Foulsham et al. 2013, 2018; Ossandón et al. 2014), those in previous studies had no horizontally flipped counterparts (e.g., Nuthmann and Matthias 2014). Not controlling for left–right differences in the scenes possibly makes it harder to detect small effects of pseudoneglect on search efficiency. By their very nature, naturalistic scenes vary considerably. Therefore, whether or not pseudoneglect affects search efficiency may also depend on the stimulus set used. To improve generalizability, it is important to account for stimulus sampling variability in statistical models (Yarkoni 2022), which is why we analyzed our data with mixed-effects models that included scene items as random effects (Nuthmann et al. 2017).

A few studies have begun to investigate whether the pseudoneglect bias during scene viewing is modulated by participant variables. For free-viewing of scenes, the leftward bias was found to be attenuated with increasing age (Chiffi et al. 2021). Regarding handedness, the evidence is contradictory. Whereas Foulsham et al. (2018) found no effect of handedness, Ossandón et al. (2014, Experiment 2) found that the pseudoneglect was almost absent for left-handers. Finally, reading direction habits appear to play a role (Afsari et al. 2016, 2018).

In one of their free-viewing experiments, Ossandón et al. (2014, Experiment 2) tested whether the pseudoneglect bias is related to the global-to-local processing of visual information (Hellige 1996; see also Poynter and Roberts 2012). Evidence from previous research suggests that the right hemisphere is more specialized for global processing, whereas the left hemisphere is more specialized for local processing (e.g., meta-analysis by Van Kleeck 1989). Based on the assumption that global processing is mediated by low spatial frequencies and local processing by higher spatial frequencies (Badcock et al. 1990; Kauffmann et al. 2014), spatial frequency filtering was applied to the scene stimuli. Whereas high-pass filtered images resemble line drawings, low-pass filtering essentially creates blurred images. If the pseudoneglect bias is due to right hemispheric dominance in the processing of global stimulus properties, it should be more pronounced for low-pass filtered scenes and less pronounced for high-pass filtered scenes, relative to unfiltered images (Ossandón et al. 2014). However, spatial frequency filtering did not significantly affect the spatiotemporal profile of fixations, suggesting that hemispheric asymmetries for global and local processing cannot explain the pseudoneglect bias. Instead, both Ossandón et al. (2014) and Nuthmann and Matthias (2014) proposed that the leftward bias in scene viewing reflects the lateralization of relevant attentional control mechanisms to the right hemisphere of the brain (Corbetta and Shulman 2002; see also Gigliotta et al. 2017).

In summary, we used eye-movement recordings to track the time course of pseudoneglect during scene search with high spatial and temporal resolution. We found that the initial leftward bias in the gaze data was accompanied by a small but significant reduction in search efficiency for right-side targets. When designing visual-search experiments, it may therefore be advisable to take spatial asymmetries into account by controlling for left–right target location or by including horizontally flipped scene versions (see Ramzaoui et al. 2021; Spotorno and Tatler 2017).

## Data Availability

Data and analysis code are available at https://osf.io/54t6g/.

## Footnotes

1 In both studies, the researchers focused on the patients and compared them with the controls, which is why no separate statistical analyses for the control group were reported.

2 When the data were averaged for each scene and then averaged across scene items (Ossandón et al. 2014), the qualitative pattern of results did not change.
